# *Twist* Controls Skeletal Development and Dorsoventral Patterning by Regulating *Runx2* in Zebrafish

**DOI:** 10.1371/journal.pone.0027324

**Published:** 2011-11-07

**Authors:** Der-Chih Yang, Chih-Chien Tsai, Yun-Feng Liao, Hui-Chuan Fu, Huey-Jen Tsay, Tung-Fu Huang, Yau-Hung Chen, Shih-Chieh Hung

**Affiliations:** 1 Institute of Clinical Medicine, National Yang-Ming University, Taipei, Taiwan; 2 Department of Pharmacology, National Yang-Ming University, Taipei, Taiwan; 3 Department of Neuroscience, National Yang-Ming University, Taipei, Taiwan; 4 Department of Surgery, National Yang-Ming University, Taipei, Taiwan; 5 Department of Chemistry, Tamkang University, Taiwan; 6 Department of Medical Research and Education, Veterans General Hospital - Taipei, Taipei, Taiwan; 7 Orthopaedics and Traumatology, Veterans General Hospital - Taipei, Taipei, Taiwan; Brigham and Women's Hospital, United States of America

## Abstract

**Background:**

Twist1a and twist1b are the principal components of twists that negatively regulate a number of cellular signaling events. Expression of *runx2* and downstream targets is essential for skeletal development and ventral organizer formation and specification in early vertebrate embryos, but what controls ventral activity of maternal *runx2* and how *twists* function in zebrafish embryogenesis still remain unclear.

**Methodology/Principal Findings:**

By studying the loss of *twist* induced by injection of morpholino-oligonucleotide in zebrafish, we found that *twist1a* and *twist1b*, but not *twist2* or *twist3*, were required for proper skeletal development and dorsoventral patterning in early embryos. Overexpression of *twist1a* or *twist1b* following mRNA injection resulted in deteriorated skeletal development and formation of typical dorsalized embryos, whereas knockdown of *twist1a* and *twist1b* led to the formation of abnormal embryos with enhanced skeletal formation and typical ventralized patterning. Overexpression of *twist1a* or *twist1b* decreased the expression of *runx2b*, whereas twist1a and twist1b knockdown increased *runx2b* expression. We have further demonstrated that phenotypes induced by *twist1a* and *twist1b* knockdown were rescued by *runx2b* knockdown.

**Conclusions/Significance:**

Together, these results suggest that twist1a and twist1b control skeletal development and dorsoventral patterning by regulating *runx2b* in zebrafish and provide potential targets for the treatment of diseases or syndromes associated with decreased skeletal development.

## Introduction

The initiation of skeletal development begins with the migration and proliferation of cells from cranial neural crest, sclerotome, and lateral plate mesoderm into mesenchymal condensations that form the template of the future skeleton [1]. Under a precise genetic regulation by a repertoire of transcription factors, chondrocytes or osteoblasts arise from these condensations to form cartilage and bone [2]. Understanding the signaling pathways involved in skeletal development will help to treat diseases associated with abnormal bone formation.

The vertebrate dorsoventral axis establishment represents the earliest event where programmes of induction and cellular commitment are used. The process is controlled by the actions of maternal and zygotic genes, and usually involves cell–cell interactions, cell movements, and spatiotemporally controlled expression of dorsoventral determinants [3], [4], [5]. The identification of target genes of the signals involved in embryonic axis development will help to establish the genetic network underlying these processes.

Zebrafish was described as ‘the canonical vertebrate’, due to the similarities between zebrafish and mammalian biology. Because of the transparent and continuous visualization of the developmental processes, the use of rapid and transient assays, and the feasibility and affordability of large-scale forward genetic screens in zebrafish [6], [7], it has attracted researchers from various fields, such as neuroscience, hematopoiesis or cardiovascular research. These shared features have prompted many laboratories to begin exploiting the unique advantages of the zebrafish system to study human disease, especially in the identification of human disease gene homologs. Thus, the zebrafish is a good model for investigating human development, including skeletal development and dorsoventral axis establishment.

Runx2 (runt-related transcription factor 2, also known as Cbfa1, Osf2 and AML3) is not only a transcription factor essential for skeletal development [8], [9], but also an important maternal determinant of ventral zygotic genes in zebrafish [10]. Previous studies have identified two orthologs of the mammalian Runx2, *runx2a* and *runx2b*, in zebrafish. Both genes have type 1 (T1) and type 2 (T2) isoforms and share sequence homology and gene structure with the mammalian genes, and map to regions of the zebrafish genome displaying conserved synteny with the region where the human gene is localized. Although both genes are expressed in developing skeletal elements and skeletal defects appeared following depletion of either *runx2a* or *2b*, the effect of *runx2b* knockdown on skeletal defects is much more significant than *runx2a* knockdown [11]. In addition, *runx2b* have shown to be able to regulate the expression of osterix and osteocalcin[12]–[13]; these observations strongly indicate the important role of *runx2b* during developmental bone formation. Moreover, depletion of maternal *runx2b* (especially T2 isoform), rather than *runx2a*, strongly dorsalizes embryos, due to loss of the earliest zygotic expression of ventral genes, resulting in expansion of dorsal gene expression [10], [14].

*Twist* genes code for regulatory bHLH proteins that are essential for embryonic development and are conserved across the metazoan [15]. The zebrafish twist family comprises four genes: *twist1a*, *twist1b*— orthologs to mammalian *Twist1*, *twist2*— ortholog to mammalian *Twist2*; and *twist3*—a gene from a new clade that does not exist in mammals [16]. Previous study has demonstrated that the twist developmental patterns, e.g., expression in cephalic neural crest, sclerotome and lateral plate mesoderm, are conserved in tetrapods to the fish [16]. *twist* is abundantly expressed in invaginating/migrating cells in jellyfish. However, the roles of *twist* in skeletal development and axis establishment in zebrafish remain to be clarified.

We have demonstrated that *TWIST*, activated by HIF-1α under hypoxic conditions, inhibited human mesenchymal stem cell (MSC) osteogenesis via direct downregulation of T2 *RUNX2,* which led to suppression of T1 *RUNX2* (DC Yang et al, 2011, PLoS ONE, In press). Since several signaling pathways involved in craniofacial skeletal development in zebrafish are similar to the pathways involved in osteogenic differentiation of mammalian MSCs [6], [17], we hypothesized that the expression of T1 and T2 *runx2b* would also be suppressed by *twist* in zebrafish under hypoxic conditions. In the current study, we first found that in zebrafish under hypoxic conditions, bone mineralization was inhibited and T1 and T2 *runx2b* and their downstream targets were downregulated, while *hif-1α* and *twist* were upregulated. Inhibition of twist1a and twist1b by morpholino oligonucleotides (MO) increased the expression of T1 and T2 *runx2b*, and induced ventralized patterning, while microinjecting zebrafish embryos with full length *twist1a* and *twist1b* mRNA decreased the expression of T1 and T2 *runx2b*, and induced dorsalized patterning in zebrafish. Twist1a and twist1b morphlinos also rescued hypoxia-induced decrease in craniofacial skeletal development in zebrafish.

## Results

### Hypoxia inhibits bone mineralization in zebrafish

**Figure 1:**
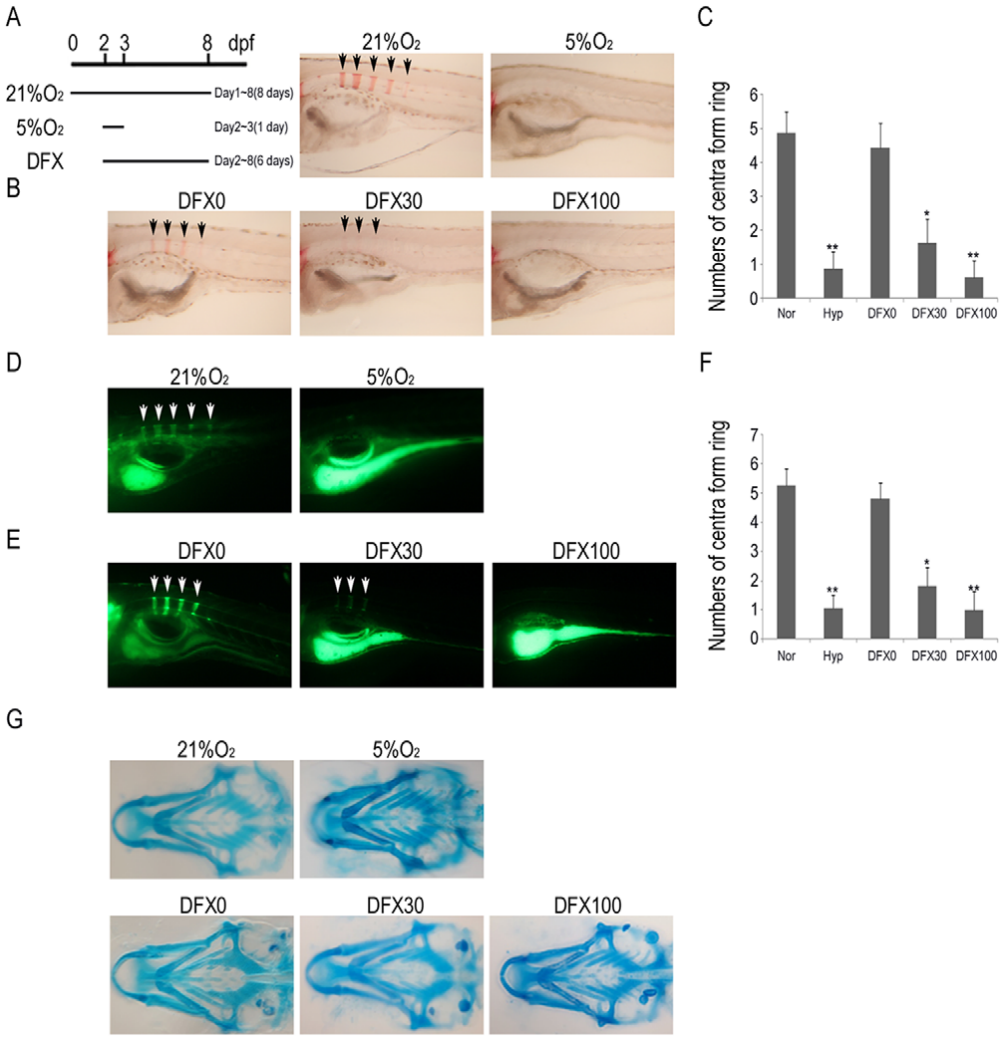
Hypoxia inhibits mineralization but not chondrogenesis in zebrafish. A, **D, G** Zebrafish at 2 days post-fertilization (dpf) were treated with 5% O_2_ for 1 day or **B**, **E, H** indicated concentration of DFX for 6 days and stained with (**A, B**) ARS, (**D, E**) calcein labeling, or (**G, H**) Alcian blue staining at 8 dpf. Mineralization was indicated by positive ARS staining or calcein labeling in the spine area (arrow) and the number of positive staining developing centra form ring with ARS or calcein labeling were calculated (C, F).

To understand the effects of hypoxia on skeletal development, zebrafish at 2 dpf (day post fertilization) were subjected to the hypoxic environment (5% O_2_) for 1 day and stained with Alizarin Red S (ARS) for determination of the degree of mineralization at 8 dpf. Hypoxia treatment induced a decrease in ARS staining in the spine area (Fig. 1A) compared to the control group. Similarly, treatment of zebrafish at 2 dpf with DFX, a hypoxia-mimicking agent, for 6 days also induced a decrease in ARS staining in the spine compared to the control group (Fig. 1B). The numbers of positive staining developing centra form ring with ARS were calculated (Fig. 1C). To further visualize normal and defective bone development in zebrafish embryos induced by hypoxia or treatment with DFX, the embryos were labeled with the fluorescent chromophore calcein. Treatment of zebrafish at 2 dpf with hypoxia (Fig. 1D) or DFX (Fig. 1E) and quatitative data (Fig. 1F) for 6 days also induced a decrease in calcein labeling in the spine area compared to the control group. To examine whether decreased bone mineralization was attributed to the reduction in chondrogenesis, Alcian Blue staining was performed at 8 dpf. At this time, no obvious reduction in Alcian Blue staining was noted in zebrafish exposed to hypoxia or DFX treatment compared to the controls (Fig. 1G and Fig. 1H). Collectively, these data suggest hypoxia or DFX treatment inhibited bone mineralization but not chondrogenesis in zebrafish.

### Hypoxia inhibits the expression of type 1 and type 2 runx2b and their downstream targets

**Figure 2:**
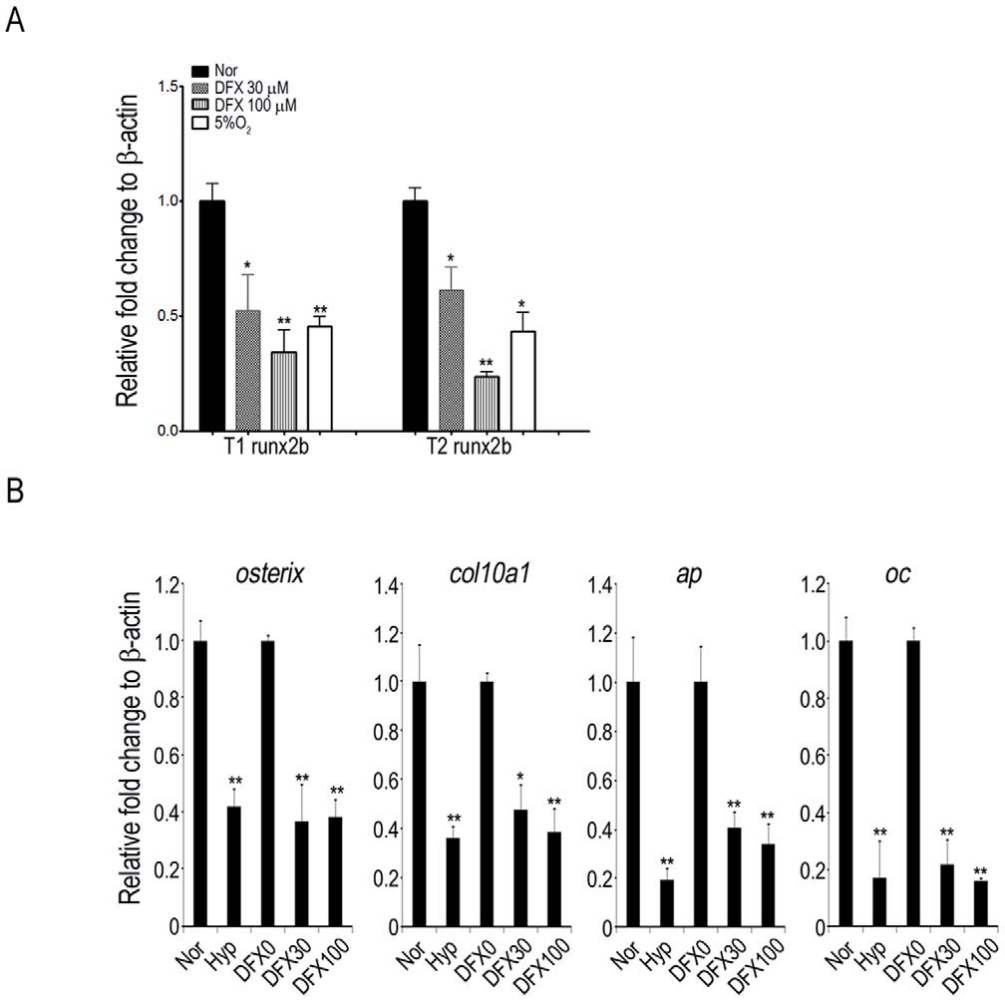
Hypoxia inhibits T1 and T2 runx2b expression in zebrafish. Zebrafish at 48 hpf were treated with 21% O_2,_ 5% O_2_ or indicated concentration of DFX for 1 day, and gene expression of T1 and T2 runx2b was assayed by quantitative RT-PCR. Results are shown as relative expression to β-actin (mean ± SD) and significance was determined by Student's t-test. (* p<0.05 and ** p<0.01 versus 21% O_2_).

Because MO-based loss-of-function studies revealed the involvement of *runx2b*, rather than *runx2a* in skeletal development [11], we chose *runx2b* for exploring the key molecule that was targeted by hypoxia or DFX to regulate osteogenesis. We found that when zebrafish was exposed to hypoxia or treated with DFX at 48 hpf for 24 h, there was decreased expression of T1 and T2 *runx2b* (Fig. 2A). Consistent with the effects on upstream transcription factor, hypoxia exposure or DFX treatment also decreased the expression of *osterix*, *collagen 10a1 (col10a1)*, *alkaline phosphatas*e (*ap*) and *osteocalcin (oc)*, (Fig. 2B). Collectively, these data suggest hypoxia or DFX treatment downregulates T1 and T2 *runx2b* and their downstream targets to inhibit skeletal development in zebrafish.

### Hypoxia downregulates type 1 and type 2 *runx2b* via the *hif-1α*-*twist* pathway

**Figure 3:**
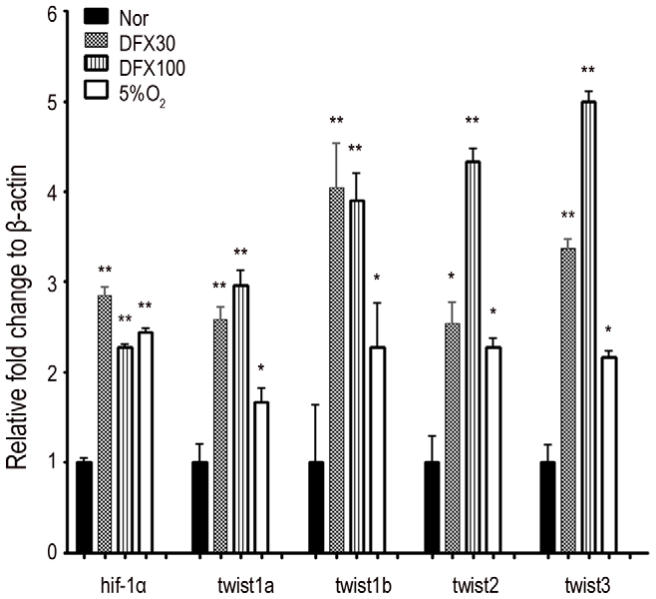
Hypoxia increases the expression of hif1-α and twist in zebrafish. Zebrafish at 48 hpf were treated with or without 21% O_2,_ 5% O_2_ or indicated concentration of DFX for 1 day and the expression of hif-1α, twist1a, twist1b, twist2 and twist3 genes was assayed by quantitative RT-PCR. Results are shown as the relative expression to β-actin (mean ± SD) and significance was determined by Student's t-test. (* p<0.05 and ** p<0.01 versus 21% O_2_).

It has been demonstrated that HIF-1α directly downregulated Twist under hypoxic conditions in mammalian cells [18]. To examine the molecular mechanism involved in hypoxia-mediated inhibition of osteogenesis, we first demonstrated that the expression of *hif1α a*nd *twists* (*twist1a*,*1b*, *2* and *3*) (Fig. 3) was increased when zebrafish was treated with DFX or hypoxia at 48 hpf for 24 h. These data suggest hypoxia inhibits skeletal development and activates the *hif1*-*twist* pathway in zebrafish.

### *twist1a* and *twist 1b* knockdown independently inhibits *runx2b* expression and induces ventralized embryos in zebrafish

**Figure 4:**
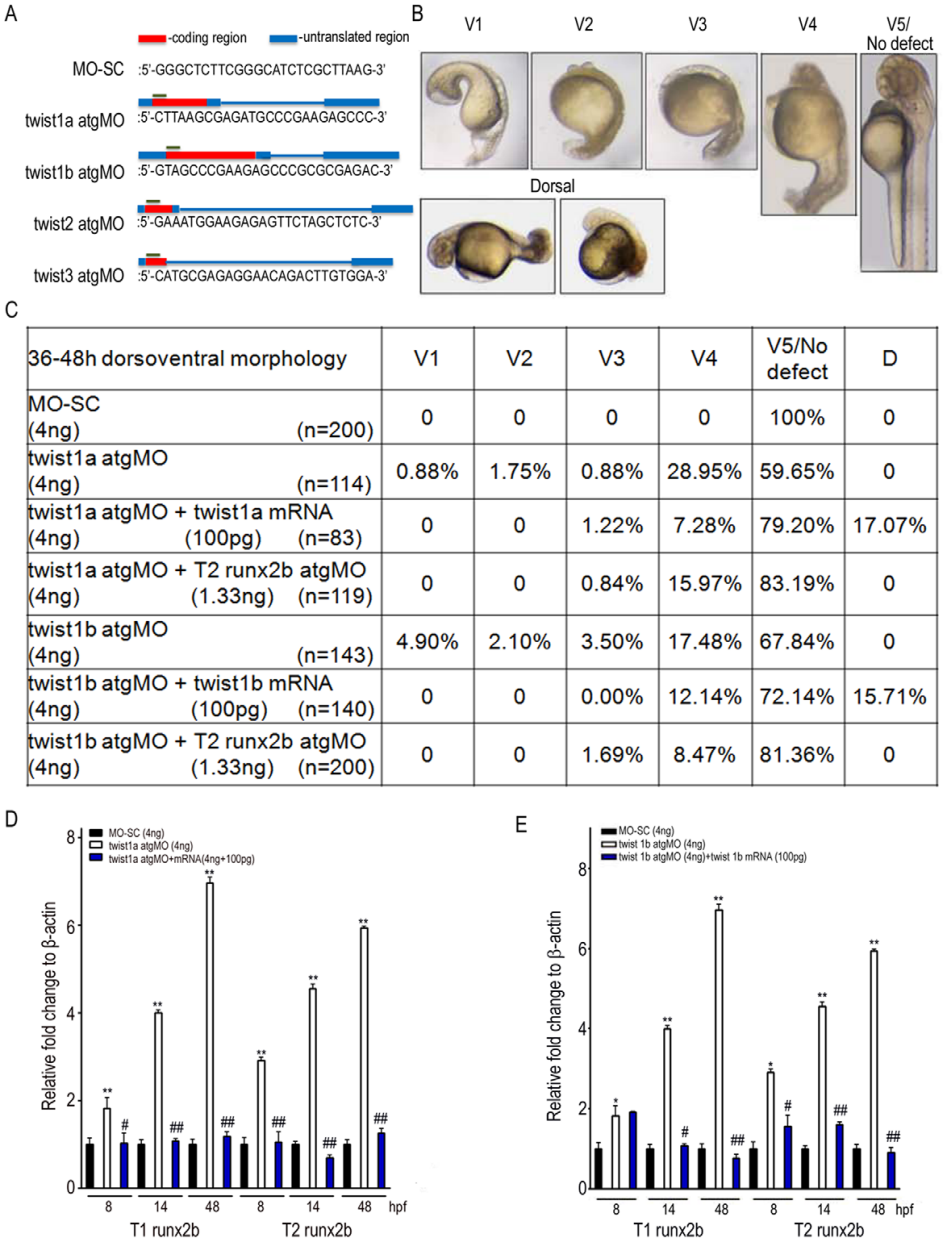
Morpholino knockdown of twist1a and twist1b induces the appearance of ventralized embryos and increases the expression of runx2b in zebrafish. **A**, Antisense morpholinos (MO) for twist1a (twist1a atgMO), twist1b (twist1b atgMO), twist2 (twist2 atgMO) and twist3 (twist3 atgMO), were designed against the 5′ UTR and ATG regions, which blocked translation of each transcript. Each atgMO was microinjected into 1-cell to 4-cell embryo and the percentage of each dorsoventral patterning was calculated at 36–48 hpf. **B**, Representative pictures of induced Class 1–5 ventralized (V1-5/no defect) and dorsalized (D) embryos. V and D phenotype annotations were described in ref. 20 and 21, respectively. **C**, Table summarizing the ventralized or dorsalized embryo features of zebrafish microinjected with indicated concentration of each atgMO with or without twist1a/1b mRNA or T2 runx2b atgMO. Zebrafish were microinjected with MO-SC, (**D**) twist1a atgMO or (**E**) twist1b atgMO with or without (**D**) twist 1a mRNA or (**E**) twist 1b mRNA and quantitative RT-PCR for T1 runx2b and T2 runx2b were performed at 8, 14 and 48 hpf (n = 3). Results are shown as the relative expression to β-actin (mean ± SD) and significance was determined by Student's t-test. (* p<0.05 and ** p<0.01 versus MO-SC; # p<0.05 and ## p<0.01 versus twist1a/1b atgMO).

Since runx2b also controls the dorsoventral patterning in the early zebrafish embryos [10], [14], we therefore examined the involvement of the twist isoforms in zebrafish axis establishment [16]. Interestingly, microinjection of twist1a and twist1b atgMOs but not scrambled MO (MO-SC), twist2 and twist3 atgMOs dose-dependently induced an increase in Class 3 (V3) and 4 (V4) ventralized embryos [19] (Fig. 4A–C, Fig. S1A), in which anterior forebrain was deficient, and eyes and notochord were completely lost. Because runx2b upregulates ventral genes [10], [14], these results suggested that knockdown of *twist1a* and *twist1b* increased the expression of *runx2b*. Consistently, quantitative RT-PCR revealed twist1a and twist1b atgMOs increased T1 and T2 *runx2b* expressions compared to MO-SC embryos at 8, 14, and 48 hpf (Fig. 4D, 4E), while MO-SC, twist2 and twist3 atgMOs failed to induce any increase in *runx2b* expression (Fig. S1B).

More importantly, twist1a or twist1b atgMO-induced ventralized embryos and increase in *runx2b* gene expression were rescued by co-injection with *twist1a* or *twist1b* mRNA (Fig.4C–4E). Further, injection of *twist1a* or *twist1b* mRNA alone induced the appearance of dorsalized embryos (Fig. S1C), in which ventral tail vein and fin were missing, blood circulation was impaired [20], and the expressions of T1 and T2 *runx2b* were inhibited (Fig. S1D). It has been reported that knockdown of T2 *runx2b* with T2 runx2b atgMO induces the appearance of dorsalized embryos [10]. Similarly, twist1a or twist1b atgMO-induced increase of the ventralized embryos were inhibited by co-injection with T2 runx2b atgMO (Fig. 4C), suggesting the involvement of T2 *runx2b* in determining the twist1a or twist1b atgMOs-induced ventralized patterning.

**Figure 5:**
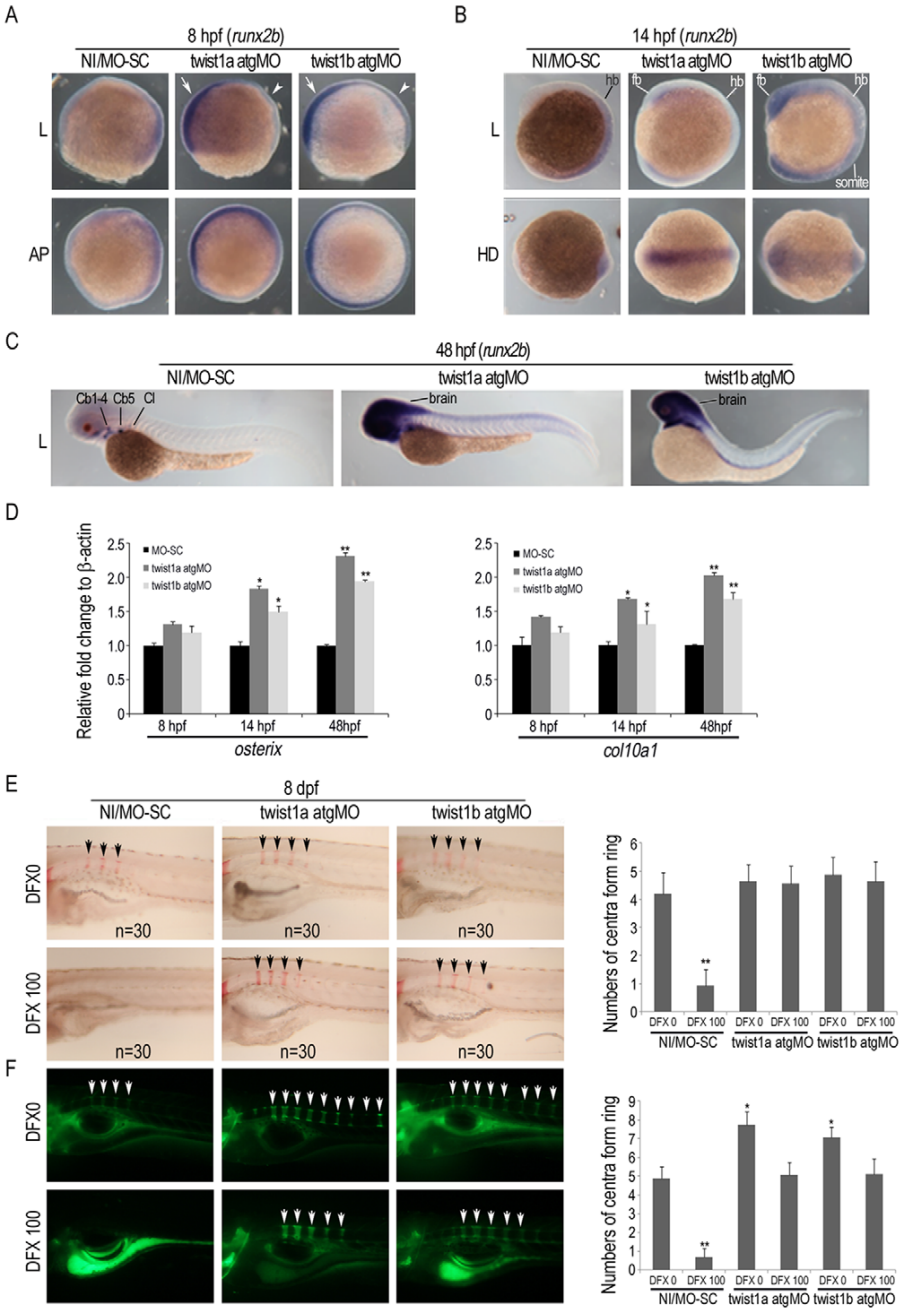
Morpholino knockdown of twist1a and twist1b induces runx2b expression and promotes bone formation in zebrafish. (A to D) Zebrafish were microinjected without (No injection, NI) or with control MO (MO-SC), twist1a or twist1b atgMOs and runx2b expression was analyzed by in situ hybridization at 8 hpf (**A**), 14 hpf (**B**), and 48 hpf (**C**) with runx2b probe. Pictures of lateral (L), animal pole (AP), and head region dorsal (HD) views are displayed. Runx2b expression was induced by twist1a or twist1b atgMO compared to MO-SC at 8 hpf, 14 hpf and 48 hpf. (arrow, ventral region; arrowhead, dorsal region; fb, forebrain area; hb, hindbrain area; ceratobranchial 1–5, Cb1-5; cleithrum, Cl) **D**, Quantitative RT-PCR for osterix and col10a1 was performed at 8, 14 and 48 hpf (n = 3). Results are shown as the relative expression to β-actin (mean ± SD) and significance was determined by Student's t-test. (* p<0.05 and ** p<0.01 versus MO-SC). **E, F,** Zebrafish survived after microinjection with MO-SC or twist1a or twist1b atgMO were cultured with or without 100 µM DFX and bone mineralization was analyzed by (**E**) ARS staining and (**F**) calcein labeling at 8 dpf. The number of positive staining developing centra form ring with ARS or calcein labeling were calculated.

Whole-mount *in situ* hybridization further demonstrated that no injection or MO-SC embryos expressed endogenous *runx2b* on all enveloping layer in a faint manner at 8 and 14 hpf, respectively (Fig. 5A, 5B). In contrast, the expression of *runx2b* at 8 hpf was strongly induced by twist1a atgMO and twist1b atgMO in the enveloping layer on the future ventral region (Fig. 5A). At 14 hpf, *runx2b* expression was induced by twist1a atgMO mainly in the neural crest of the forebrain and by twist1b atgMO both in the neural crest of the forebrain and somites (Fig. 5B). At 48 hpf, twist1a atgMO increased *runx2b* expression in the whole brain and produced new signals in the neural tube region of the V5/no defect embryos compared to no injection zebrafish, embryos injected with MO-SC, twist2 or twist3 atgMOs (Fig. S2), which only showed *runx2b* expression in ceratobranchial 1–5 (Cb1-5) and cleithrum (Cl); twist1b atgMO also increased *runx2b* expression in the whole brain and produced a new signal in the tail of the V5/no defect embryos (Fig. 5C).

### *twist1a* and *twist1b* knockdown independently enhances bone formation in zebrafish

We then examined whether microinjection of twist1a and twist1b atgMOs upregulated the expression of other osteoblast markers. Quantitative RT-PCR revealed both twist1a and twist1b atgMOs enhanced the expression of *osterix* and *col10a1* at 14 and 48 hpf, while no significant change was noted at 8 hpf. (Fig. 5D). Finally, we determined whether microinjection of twist1a and twist1b atgMOs promoted functional mineralization both under normoxia and hypoxia. The embryos had normal morphology and survived up to 8 dpf were assayed for the degree of mineralization. These embryos showed increase in bone mineralization, as evident by the apparent increase of ARS staining in the spine area (Fig. 5E) in whole embryos studies. Similarly, calcein labeling also revealed an increase in bone mineralization in embryos microinjected with twist1a and twist1b atgMOs both in the absence or presence of DFX treatment (Fig. 5F). Together, these data suggest that the knockdown of *twist1a* or *twist1b* in zebrafish enhanced *runx2b* transcription, induced ventralized patterning, and promoted bone formation both under normoxic and hypoxic conditions.

## Discussion

Twist is known to trigger epithelial-mesenchymal transition (EMT) mechanisms and increase cells with migratory ability. Although Twist is constantly expressed in various cells including osteoblasts, its roles in skeleton development is seldom, if ever, investigated [21]. Previous studies have shown that TWIST silencing enhanced osteoblast gene expression and matrix mineralization [22], [23]. In this current study, we demonstrate that twist plays an essential role in skeleton development and axis establishment by regulating *runx2b* in zebrafish. Interestingly, only *TWIST* in human MSCs and *twist1a* and *twist1b* (orthologs of mammalian *Twist*), but not *twist2* and *twist3*, in zebrafish possess these functions. Moreover, *RUNX2* in human and *runx2b* in zebrafish are mainly involved in skeleton development or axis establishment, suggesting the conservation of Twist-Runx2 pathway in mammalian cells and zebrafish.

Previously, Twist has been demonstrated to inhibit DNA binding and gene activation by Runx2, while *Runx2* expression was not affected in mice carrying Twist heterozygosity [24]. The current study found overexpression and knockdown of *Twist* increased and decreased the expression of *Runx2* both at the mRNA and protein levels, respectively. The discrepancy between our study and the previous one [24] may be because the suppression of *Twist* expression by Twist heterozygosity in mice is not sufficient to downregulate *Runx2*, as is the minimal effect of the low dose of twist1b atgMO to increase *runx2b* expression (Fig. S3).

Twist has been reported to have a synergistic effect with dorsal and snail in integrating diverse dorsoventral patterning in the Drosophila embryo [25], [26]. Runx2b, a maternal and zygotic mediator, has been reported to induce the expression of ventral gene such as *ved*, *vent* and *vox* in the earliest embryo of zebrafish (4 hpf) [10]; however the upstream molecule regulating the expression of *runx2b* has not been discovered. Knockdown of *twist1a* and *twist1b* inducing the expression of *runx2b* was observed as early as 8 hpf and 14 hpf, suggesting the early involvement of *twist* in controlling the dorsoventral patterning. The phenotypes of the knockdown of *twist1a* and *twist1b* include abnormalities in eyes, fusion of fore/midbrain and hindbrain, notochord, trunk, and other skeleton deformity, which are normally observed in the ventralized embryos induced by mutation or knockdown of dorsal-specific genes [19] or overexpression of ventral-specific genes [27].

Together with Runx2, a number of molecules involved in dorsoventral patterning also participate in bone formation, including BMPs, Chordin, Noggin, Wnt/β-catenin, transforming growth factor-β (TGF-β) and fibroblast growth factors (FGFs) [28], [29]. Runx1 and Runx3 also interact with similar molecules in haematopoiesis and gastric epithelial maintenance, respectively [30], [31]. Whether the expressions of these genes are also regulated by twist has not been clarified and necessitates future investigation. Future exploration of the twist signaling pathways may help in developing strategies to control skeleton development and dorsoventral patterning through suppressing runx2b.

In conclusion, these data provide convincing evidences for the important roles of Twist in controlling dorsoventral patterning, skeleton development and bone mineralization. Further exploration of the mechanism involved in Twist-mediate regulation of skeleton development and regeneration may provide new strategies for treating these diseases.

## Methods

### Zebrafish maintenance and histological staining: Alcian blue and ARS staining

The procedures of ARS stain are described by Walker and Kimmel [32]. Zebrafish (Danio rerio) embryos were maintained in Embryo Medium at 28.5°C. To visualize developing bone at 8dpf, the embryos were immobilized in ice water, and then fixed for 24 h in buffered 4% paraformaldehyde. After washing twice with PBS, they were dehydrated in 50% and 100% ethanol each for 24 h. They were then placed in 0.05 mg/ml Alcian Blue in 3∶7 glacial acetic acid: 100% ethanol for 20 min. Alcian Blue-stained zebrafish were then cleared in 1% KOH for 1 h. The fish were then transferred to 2 mg/ml Alizarin Red S in 1% KOH for 1 h and cleared in 20% glycerol in 1% KOH for 40 min. Alizarin Red S-stained zebrafish were photographed or preserved in solution made by 4 parts 100% glycerol plus 1 part 95% ethanol (v:v).

### Calcein immersion

The procedures of calcein immersion are described by Du *et al.* [33] and Chen et al. [34] with some modifications. Briefly, 0.2% (w/v) of calcein (Sigma) solution was prepared and adjusted to pH 7.0 with 0.5 N NaOH. Zebrafish embryos from 8 day were immersed in the calcein solution in petri dishes for 5 min. Calcein-immersed embryos were rinsed several times with tap water, and then immersed in one liter of tap water for 10 min to allow the excess calcein to diffuse out of the tissues. The embryos were then euthanized in tricaine–methanesulfonate (MS 222), mounted on glass slides with methyl-cellulose (3%), and observed under a fluorescent microscopy.

### RNA extraction and quantitative RT-PCR

Total RNA was prepared using the Trizol reagent (Invitrogen) according to the manufacturer's specifications. cDNA was synthesized from 2 µg RNA and using Superscript III (Invitrogen), random primers (Invitrogen), 10 mM DTT (Invitrogen), and RNaseOUT ribonuclease RNase inhibitor (Invitrogen). cDNA was then 1/20 diluted with ddH_2_O (Final cDNA concentration: 5 ng/µl). The quantitative RT-PCR was performed using 40 ng cDNA as the template in a 20 µl reaction mixture containing FastStart SYBR Green Master (Roche Applied Science) and a specific primer pair of each cDNA according to the published sequences which listed in the Table S1. Analysis of the results were carried out using the software supplied with the ABI Step One Real-Time PCR System machine and each expression was calculated relative to the zebrafish β-Actin (delta CT) and then relative to controls (delta delta CT) using the fluorescence threshold of the amplification reaction and the comparative CT method.

### Preparation and microinjection of morpholino or RNA

Twists Morpholino antisense oligonucleotides (twist-MOs) were obtained from Gene Tools (Philomath, OR) with the sequences: zebrafish twist1a atgMO (5′-CTTAAGCGAGATGCCCGAAGAGCCC-3′); twist1b atgMO (5′-GATGCCCGAAGAGCCCGCGCGAGAC-3′); twist2 atgMO (5′-GAAATGGAAGAGAGTTCTAGCTCTC-3′); twist3 atgMO (5′-CATGCGAGAGGAACAGACTTGTGGA-3′) and T2 runx2b atgMO (5′-CATGGTCGCCACTTTCGCTCCCAAA-3′) targets the sequence at the translation start site of twist mRNAs. As a control experiment, Scrambled control (MO-SC, 5′-GGGCTCTTCGGGCATCTCGCTTAAG-3′) recommended by Gene Tools was used. All of the above were prepared at stocking concentrations of 1 mM and 2.3 nl per embryo was injected. Synthetic capped-mRNAs were prepared using mMessage mMachine (Ambion) from pGEMT-twist1a or pGEMT-twist1b, which containing coding sequences of zebrafish twist1a and twist1b, respectively. Embryos were collected by natural spawning and microinjected with MO or mRNA (overexpression) or MO/mRNA mixtures (rescue experiments) at 1- or 8-cell stage using a Narishige micromanipulator. For constructing plasmids pGEMT-twist1a and pGEMT-twist1b, the pDNR-LIB-twist1a and pME18s-FL3-twist1b were purchased from Open Biosystems and zebrafish twist1a or twist1b mRNAs were amplified by PCR using the primer pairs: twist1a-rescue-F: 5′-GATGTTCGAAGAGGAAGCCAT-3′; twist1a-rescue-R: TTTCCTGCAGCGAGTCTCTGT; twist1b-rescue-F: GAGATGCCGGAGGAACCGGCCCGA; twist1b-rescue-R: AGCTTTGTATTTGCACAGGATTCG. Double underlined nucleotides indicate the modified nucleotides. The PCR products (791 and 895bp) were then subcloned into the pGEMT-easy vector (Promega).

### Whole-Mount in Situ Hybridization and Sections

Whole mount *in situ* hybridization for the runx2b gene was performed at 68°C as described by Flores *et al.*, 2008[10]. Samples were imaged using a Leica MZ16FA stereomicroscope with a Leica DC490 camera and the associated software. The designation of developmental stage of zebrafish was following those of Kimmel *et al* [35].

## Supporting Information

Figure S1 **Morpholino knockdown of twist1a and twist1b, but not twist2 and twist3, induces the appearance of ventralized embryos and increases the expression of runx2b in zebrafish.** **A,** The table summarizing the ventralized embryo features (V1-5/no defect) of zebrafish injected with indicated concentration of each atgMO. **B,** Zebrafish were microinjected with twist2 or twist3 atgMO and quantitative RT-PCR for T1 runx2b and T2 runx2b were performed at 48 hpf (n = 3). Results are shown as the relative expression to β-actin (mean ± SD). **C, D,** Microinjection of twist1a and twist1b mRNA induces the appearance of dorsalized embryos and decreases the expression of runx2b. **C,** The table summarizing the dorsalized embryo features (D1-5/no defect) of zebrafish injected with each mRNA. Dorsalized (D) phenotype annotations were described in ref. 21. **D,** Zebrafish were microinjected with or without (WT) twist1a or twist1b mRNA and quantitative RT-PCR for T1 runx2b and T2 runx2b was performed at 48 hpf (n = 3). Results are shown as the relative expression to β-actin (mean ± SD) and significance was determined by Student's t-test. (* p<0.05 and ** p<0.01 versus no injection (NI). Results are shown as the relative expression to β-actin (mean ± SD) and significance was determined by Student's t-test. (* p<0.05 and ** p<0.01 versus MO-SC).
(TIF)

Figure S2 **Morpholino knockdown of twist2 or twist3 induces no changes in runx2b transcription.** Zebrafish were microinjected with twist2 and twist3 atgMOs and runx2b expression was analyzed by in situ hybridization at 48 hpf (n = 40 for each). Pictures of lateral (L) and dorsal views (D) are displayed.
(TIF)

Figure S3 **Morpholino knockdown of twist1b increases runx2b transcription.** Morpholino knockdown of twist1b increases runx2b expression in a dose-dependent manner. Zebrafish were microinjected with indicated amount of MO-SC or twist1b atgMO and quantitative RT-PCR for T1 runx2b and T2 runx2b was performed at 48 hpf (n = 3). Results are shown as the relative expression to β-actin (mean ± SD) and significance was determined by Student's t-test. (* p<0.05 and ** p<0.01 versus MO-SC).
(TIF)

Table S1 **PCR primer list.** Primers designed to perform quantitative RT-PCR for this study are listed.
(PDF)

## References

[1] Olsen BR, Reginato AM, Wang W (2000). Bone development.. Annu Rev Cell Dev Biol.

[2] Yang X, Karsenty G (2002). Transcription factors in bone: developmental and pathological aspects.. Trends Mol Med.

[3] Heasman J (2006). Patterning the early Xenopus embryo.. Development.

[4] Wang H, Dey SK (2006). Roadmap to embryo implantation: clues from mouse models.. Nat Rev Genet.

[5] Schier AF, Talbot WS (2005). Molecular genetics of axis formation in zebrafish.. Annu Rev Genet.

[6] Yang DC, Tsay HJ, Lin SY, Chiou SH, Li MJ (2008). cAMP/PKA regulates osteogenesis, adipogenesis and ratio of RANKL/OPG mRNA expression in mesenchymal stem cells by suppressing leptin.. PLoS ONE.

[7] Lieschke GJ, Currie PD (2007). Animal models of human disease: zebrafish swim into view.. Nat Rev Genet.

[8] Ducy P, Zhang R, Geoffroy V, Ridall AL, Karsenty G (1997). Osf2/Cbfa1: a transcriptional activator of osteoblast differentiation.. Cell.

[9] Komori T, Yagi H, Nomura S, Yamaguchi A, Sasaki K (1997). Targeted disruption of Cbfa1 results in a complete lack of bone formation owing to maturational arrest of osteoblasts.. Cell.

[10] Flores MV, Lam EY, Crosier KE, Crosier PS (2008). Osteogenic transcription factor Runx2 is a maternal determinant of dorsoventral patterning in zebrafish.. Nat Cell Biol.

[11] Flores MV, Lam EY, Crosier P, Crosier K (2006). A hierarchy of Runx transcription factors modulate the onset of chondrogenesis in craniofacial endochondral bones in zebrafish.. Dev Dyn.

[12] Pinto JP, Conceicao NM, Viegas CS, Leite RB, Hurst LD (2005). Identification of a new pebp2alphaA2 isoform from zebrafish runx2 capable of inducing osteocalcin gene expression in vitro.. J Bone Miner Res.

[13] Li N, Felber K, Elks P, Croucher P, Roehl HH (2009). Tracking gene expression during zebrafish osteoblast differentiation.. Dev Dyn.

[14] Flores MV, Tsang VW, Hu W, Kalev-Zylinska M, Postlethwait J (2004). Duplicate zebrafish runx2 orthologues are expressed in developing skeletal elements.. Gene Expr Patterns.

[15] Atchley WR, Fitch WM (1997). A natural classification of the basic helix-loop-helix class of transcription factors.. Proc Natl Acad Sci U S A.

[16] Germanguz I, Lev D, Waisman T, Kim CH, Gitelman I (2007). Four twist genes in zebrafish, four expression patterns.. Dev Dyn.

[17] Hong JH, Hwang ES, McManus MT, Amsterdam A, Tian Y (2005). TAZ, a transcriptional modulator of mesenchymal stem cell differentiation.. Science.

[18] Yang MH, Wu MZ, Chiou SH, Chen PM, Chang SY (2008). Direct regulation of TWIST by HIF-1alpha promotes metastasis.. Nat Cell Biol.

[19] Kelly C, Chin AJ, Leatherman JL, Kozlowski DJ, Weinberg ES (2000). Maternally controlled (beta)-catenin-mediated signaling is required for organizer formation in the zebrafish.. Development.

[20] Mullins MC, Hammerschmidt M, Kane DA, Odenthal J, Brand M (1996). Genes establishing dorsoventral pattern formation in the zebrafish embryo: the ventral specifying genes.. Development.

[21] Xiao ZS, Thomas R, Hinson TK, Quarles LD (1998). Genomic structure and isoform expression of the mouse, rat and human Cbfa1/Osf2 transcription factor.. Gene.

[22] Kronenberg HM (2004). Twist genes regulate Runx2 and bone formation.. Dev Cell.

[23] Miraoui H, Severe N, Vaudin P, Pages JC, Marie PJ (2010). Molecular silencing of Twist1 enhances osteogenic differentiation of murine mesenchymal stem cells: implication of FGFR2 signaling.. J Cell Biochem.

[24] Bialek P, Kern B, Yang X, Schrock M, Sosic D (2004). A twist code determines the onset of osteoblast differentiation.. Dev Cell.

[25] Simpson P (1983). Maternal-Zygotic Gene Interactions during Formation of the Dorsoventral Pattern in Drosophila Embryos.. Genetics.

[26] Zeitlinger J, Zinzen RP, Stark A, Kellis M, Zhang H (2007). Whole-genome ChIP-chip analysis of Dorsal, Twist, and Snail suggests integration of diverse patterning processes in the Drosophila embryo.. Genes Dev.

[27] Rui Y, Xu Z, Xiong B, Cao Y, Lin S (2007). A beta-catenin-independent dorsalization pathway activated by Axin/JNK signaling and antagonized by aida.. Dev Cell.

[28] Frajese GV, De Martino MU, Calcagni E, Pastore R, Caprio M (2005). The epidemiology of partialandrogen deficiency in aging men (PADAM).. J Endocrinol Invest.

[29] Canalis E, Economides AN, Gazzerro E (2003). Bone morphogenetic proteins, their antagonists, and the skeleton.. Endocr Rev.

[30] Blyth K, Cameron ER, Neil JC (2005). The RUNX genes: gain or loss of function in cancer.. Nat Rev Cancer.

[31] Ito Y, Miyazono K (2003). RUNX transcription factors as key targets of TGF-beta superfamily signaling.. Curr Opin Genet Dev.

[32] Walker MB, Kimmel CB (2007). A two-color acid-free cartilage and bone stain for zebrafish larvae.. Biotech Histochem.

[33] Du SJ, Frenkel V, Kindschi G, Zohar Y (2001). Visualizing normal and defective bone development in zebrafish embryos using the fluorescent chromophore calcein.. Dev Biol.

[34] Chen YH, Lin YT, Lee GH (2009). Novel and unexpected functions of zebrafish CCAAT box binding transcription factor (NF-Y) B subunit during cartilages development.. Bone.

[35] Schilling TF, Kimmel CB (1997). Musculoskeletal patterning in the pharyngeal segments of the zebrafish embryo.. Development.

