# Supplementary figures and images for: Twist Controls Skeletal Development and Dorsoventral Patterning by Regulating Runx2 in Zebrafish
Source: PLoS One. 2011 Nov 7;6(11):e27324. doi: 10.1371/journal.pone.0027324 (PMC3210159; doi:10.1371/journal.pone.0027324)

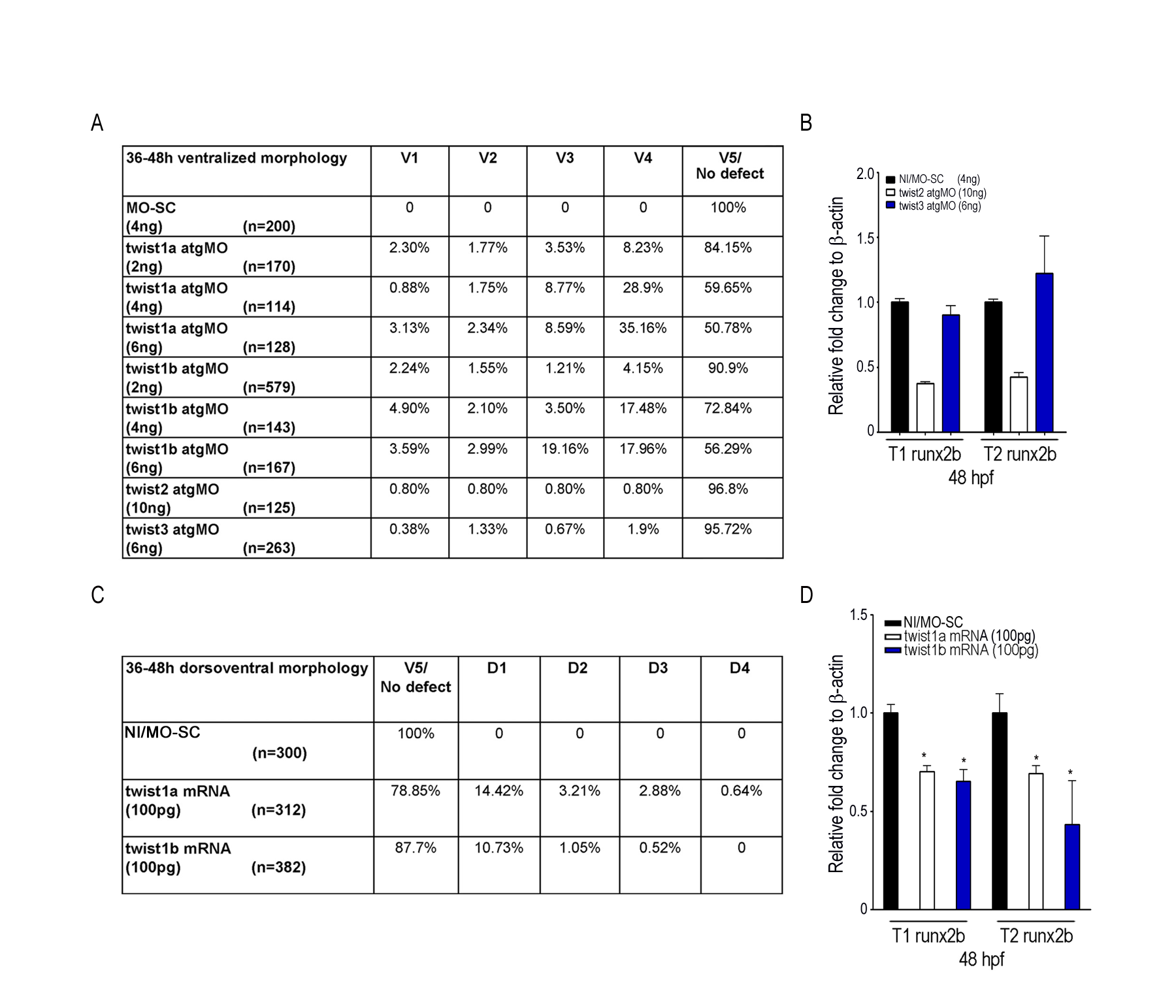

Supplement: Figure S1 — Morpholino knockdown of twist1a and twist1b, but not twist2 and twist3, induces the appearance of ventralized embryos and increases the expression of runx2b in zebrafish. A, The table summarizing the ventralized embryo features (V1-5/no defect) of zebrafish injected with indicated concentration of each atgMO. B, Zebrafish were microinjected with twist2 or twist3 atgMO and quantitative RT-PCR for T1 runx2b and T2 runx2b were performed at 48 hpf (n = 3). Results are shown as the relative expression to β-actin (mean ± SD). C, D, Microinjection of twist1a and twist1b mRNA induces the appearance of dorsalized embryos and decreases the expression of runx2b. C, The table summarizing the dorsalized embryo features (D1-5/no defect) of zebrafish injected with each mRNA. Dorsalized (D) phenotype annotations were described in ref. 21. D, Zebrafish were microinjected with or without (WT) twist1a or twist1b mRNA and quantitative RT-PCR for T1 runx2b and T2 runx2b was performed at 48 hpf (n = 3). Results are shown as the relative expression to β-actin (mean ± SD) and significance was determined by Student's t-test. (* p<0.05 and ** p<0.01 versus no injection (NI). Results are shown as the relative expression to β-actin (mean ± SD) and significance was determined by Student's t-test. (* p<0.05 and ** p<0.01 versus MO-SC). (TIF) [file pone.0027324.s001.tif]

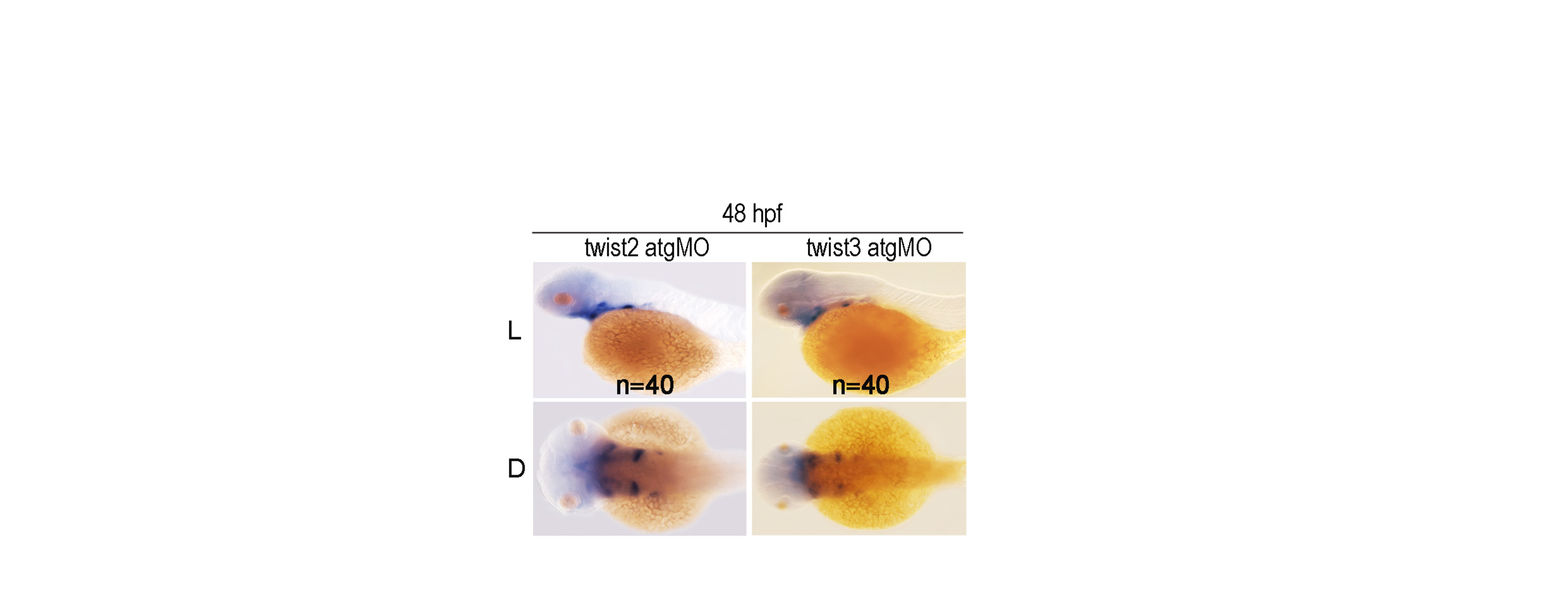

Supplement: Figure S2 — Morpholino knockdown of twist2 or twist3 induces no changes in runx2b transcription. Zebrafish were microinjected with twist2 and twist3 atgMOs and runx2b expression was analyzed by in situ hybridization at 48 hpf (n = 40 for each). Pictures of lateral (L) and dorsal views (D) are displayed. (TIF) [file pone.0027324.s002.tif]

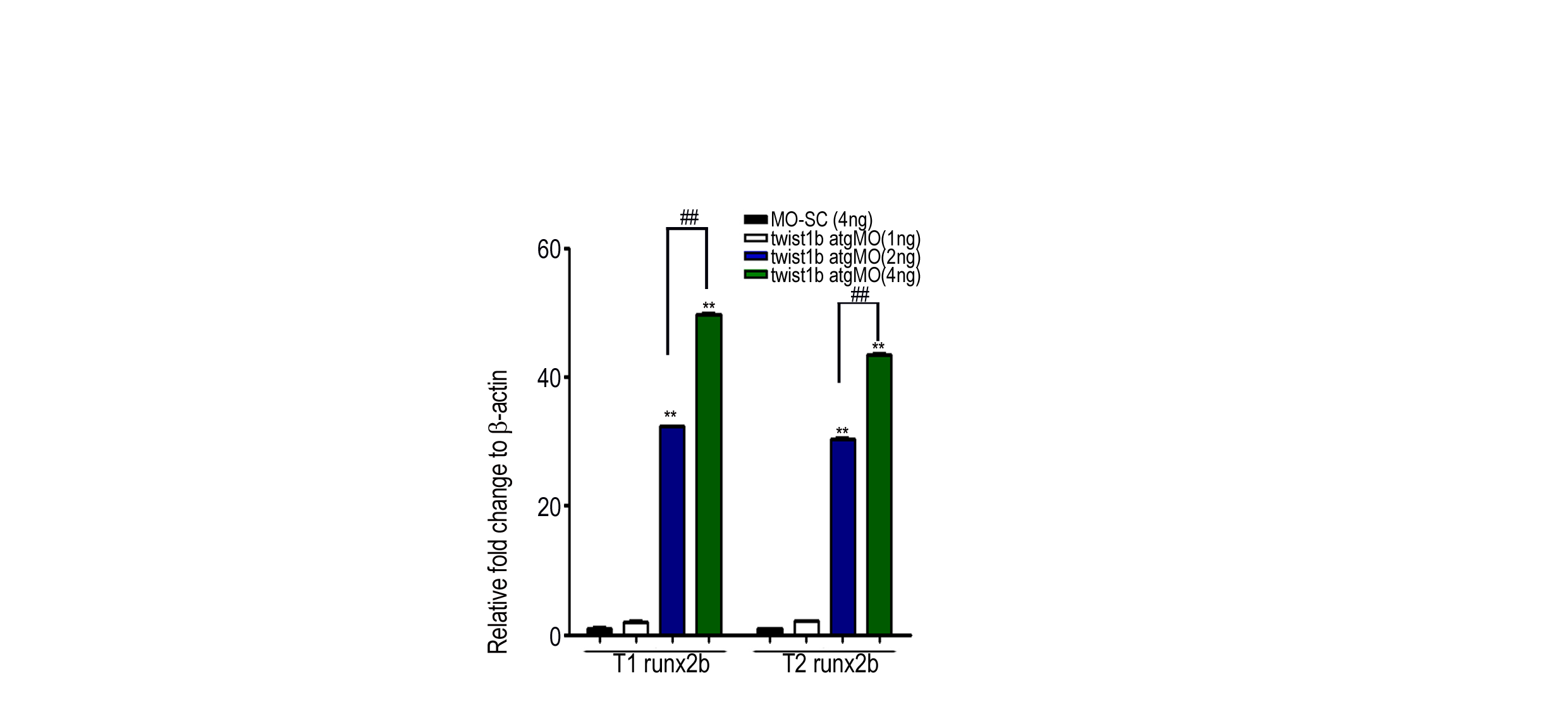

Supplement: Figure S3 — Morpholino knockdown of twist1b increases runx2b transcription. Morpholino knockdown of twist1b increases runx2b expression in a dose-dependent manner. Zebrafish were microinjected with indicated amount of MO-SC or twist1b atgMO and quantitative RT-PCR for T1 runx2b and T2 runx2b was performed at 48 hpf (n = 3). Results are shown as the relative expression to β-actin (mean ± SD) and significance was determined by Student's t-test. (* p<0.05 and ** p<0.01 versus MO-SC). (TIF) [file pone.0027324.s003.tif]
