# Supplementary material for: Twist Controls Skeletal Development and Dorsoventral Patterning by Regulating Runx2 in Zebrafish
Source: PLoS One. 2011 Nov 7;6(11):e27324. doi: 10.1371/journal.pone.0027324 (PMC3210159; doi:10.1371/journal.pone.0027324)
Supplement: Table S1 — PCR primer list. Primers designed to perform quantitative RT-PCR for this study are listed. (PDF) [file pone.0027324.s004.pdf]

Table S1 Name and sequence of primer

Name	Sequence	Size	Accession number
β-actin	F: CAACAGGGAAAAGATGACACAGAT	74	AF025305.1
	R: CAGCCTGGATGGCAACGT		
T1 runx2b	F: ACGCAA ACGGAGGACATACG	79	AY443097.1
	R: CCGGCGCTGGGATCTAC		
T2 runx2b	F: AACCACGGAGAGGCCAAAT	77	AY443096.1
	R: GGG GTCACAGAACTA AACAGGCT		
osterix	F: GCGGCATCTATATTGGAGGA	103	NM_212863.1
	R: AATCTCGGACTGGACTGGTG		
col10a1	F: CCCATCCACATCACATCAAA	103	NM_001083827.1
	R: GCGTGCATTTCTCAGAACAA		
oc	F: ACTGCACCTGGAGACCTGAC	114	NM_001083857.1
	R: TTTATAGGCGGCGATGATTC		
ap	F: ATGGGATGGGTGTTCCTACA	121	NM_201007.1
	R: GTCTTAGAGAGGGCGACGTG		
